# Cannabidiol—from Plant to Human Body: A Promising Bioactive Molecule with Multi-Target Effects in Cancer

**DOI:** 10.3390/ijms20235905

**Published:** 2019-11-25

**Authors:** Brigitta Kis, Feng Chen Ifrim, Valentina Buda, Stefana Avram, Ioana Zinuca Pavel, Diana Antal, Virgil Paunescu, Cristina Adriana Dehelean, Florina Ardelean, Zorita Diaconeasa, Codruta Soica, Corina Danciu

**Affiliations:** 1 Department of Pharmacognosy, University of Medicine and Pharmacy “Victor Babeş“, Eftimie Murgu Square, No. 2, 300041 Timişoara, Romania; kis.brigitta@umft.ro (B.K.); stefana.avram@umft.ro (S.A.); ioanaz.pavel@umft.ro (I.Z.P.); corina.danciu@umft.ro (C.D.); 2 Centre for Gene and Cellular Therapies in the Treatment of Cancer- OncoGen, Clinical County Hospital of Timişoara, Liviu Rebreanu Blvd. 156, 300736 Timişoara, Romania; vpaunescu@umft.ro; 3 Department of Marketing, medical technology, Carol Davila University of Medicine and Pharmacy, 020021 Bucharest, Romania; 4 Department of Pharmacology and Clinical Pharmacy, “Victor Babes” University of Medicine and Pharmacy, Eftimie Murgu Square, No. 2, 300041 Timisoara, Romania; 5 Department of Pharmaceutical Botany, University of Medicine and Pharmacy “Victor Babeş“, Eftimie Murgu Square, No. 2, 300041 Timişoara, Romania; diana.antal@umft.ro (D.A.); ardelean.florina@umft.ro (F.A.); 6 Department of Functional Sciences, Faculty of Medicine, “Victor Babes” University of Medicine and Pharmacy, Eftimie Murgu Square, No. 2, 300041 Timisoara, Romania; 7 Department of Toxicology, “Victor Babeş“University of Medicine and Pharmacy, Eftimie Murgu Square, No. 2, 300041 Timişoara, Romania; cadehelean@umft.ro; 8 Department of Food Science and Technology, Faculty of Food Science and Technology, University of Agricultural Science and Veterinary Medicine, Calea Manastur, 3-5, 400372 Cluj-Napoca, Romania; zorita.diaconeasa@gmail.com; 9 Department of Pharmaceutical Chemistry, University of Medicine and Pharmacy “Victor Babeş“, Eftimie Murgu Square, No. 2, 300041 Timişoara, Romania; codrutasoica@umft.ro

**Keywords:** cannabidiol, pharmacological and toxicological profile, breast cancer, prostate cancer, glioma, colon cancer, lung cancer, brain cancer, melanoma, immunomodulatory effects, clinical evidence

## Abstract

*Cannabis sativa* L. is a plant long used for its textile fibers, seed oil, and oleoresin with medicinal and psychoactive properties. It is the main source of phytocannabinoids, with over 100 compounds detected so far. In recent years, a lot of attention has been given to the main phytochemicals present in *Cannabis sativa* L., namely, cannabidiol (CBD) and Δ9-tetrahydrocannabinol (THC). Compared to THC, CBD has non-psychoactive effects, an advantage for clinical applications of anti-tumor benefits. The review is designed to provide an update regarding the multi-target effects of CBD in different types of cancer. The main focus is on the latest in vitro and in vivo studies that present data regarding the anti-proliferative, pro-apoptotic, cytotoxic, anti-invasive, anti-antiangiogenic, anti-inflammatory, and immunomodulatory properties of CBD together with their mechanisms of action. The latest clinical evidence of the anticancer effects of CBD is also outlined. Moreover, the main aspects of the pharmacological and toxicological profiles are given.

## 1. Introduction

*Cannabis sativa* L. is a plant long used for its textile fibers, seed oil, and oleoresin, with medicinal and psychoactive properties [1]. It is considered the oldest cultivated fiber plant, originating from Southeast and Central Asia [2]. Taxonomic controversies surrounding hemp have not yet resolved the issue of the *Cannabis* genus being monotypic, that is, including only one highly variable species, *Cannabis sativa* L., or polyspecific—enclosing four (*Cannabis sativa*, *Cannabis indica*, *Cannabis ruderalis*, and *Cannabis afghanica*) species with distinct geographical, chemotypic, and morphological features [3,4]. The current tendency converges towards the recognition of chemovars as the most appropriate denomination. This trend is supported by widespread crossbreeding and hybridization, as breeding barriers among *Cannabis* hybrids are absent. On the other hand, the common use of the appellation “strains” is considered improper, as this designation only applies to bacteria and viruses [5]. From a medicinal viewpoint, a demarcation of a fiber-type hemp (containing high levels of cannabidiol (CBD) but very low in psychotropic Δ9-tetrahydrocannabinol (THC) and of a drug-type *Cannabis* (containing up to 15% THC in the female inflorescences) can be made [6,7].

In Europe, great attention has been paid to the medical use of cannabis since 1840, and this was due to William O’Shaughnessy, an Irish physician who traveled to India and noticed the medicinal properties of Indian cannabis [8]. His experiments referred to cannabis use in epilepsy, tetanus, rheumatism, and cholera [9]. Later, various cannabis preparations (tinctures, extracts, cigarettes) were used in the treatment of migraines, asthma, insomnia, and even for opium-use withdrawal [8]. Despite its popularity, at the end of the 19^th^ century great variability in opinion on the therapeutic effects and preparations and also worries about drug abuse emerged [10]. The decline in use was due to the association of cannabis with addiction, mental deterioration, and crime [11], and to the replacement of cannabis preparations with synthetic drugs. This led to an international prohibition of cannabis use [11].

The identification of the major cannabinoids, THC and CBD, has been an important step for further research. Numerous studies have been conducted on THC after its isolation and characterization in the 1960s [12]. The cannabinoid receptors and the endocannabinoid system were discovered only in the 1990s, and this determined not only the evaluation of the pharmacological effects of phytocannabinoids, but also the synthesis of drugs that act on the endocannabinoid system [10].

Phytocannabinoids are a type of cannabimimetic compound which can interact with the endocannabinoid system [13]. *Cannabis sativa* L. is the main source of phytocannabinoids, with over 100 compounds detected so far [14]. The compounds accumulate in secretory hairs situated chiefly on the bracts of pistillate (female) flowers. Three different types of such trichomes have been described: bulbous glands, capitate-sessile glands, and capitate-stalked glands, resulting in a layered complex [15]. The capitate-stalked type glands contain the highest number of cannabinoids, and the biosynthesis of tetrahydrocannabinolic acid (THCA) by glandular cells has reliably been proven [16,17].

**Figure 1:**
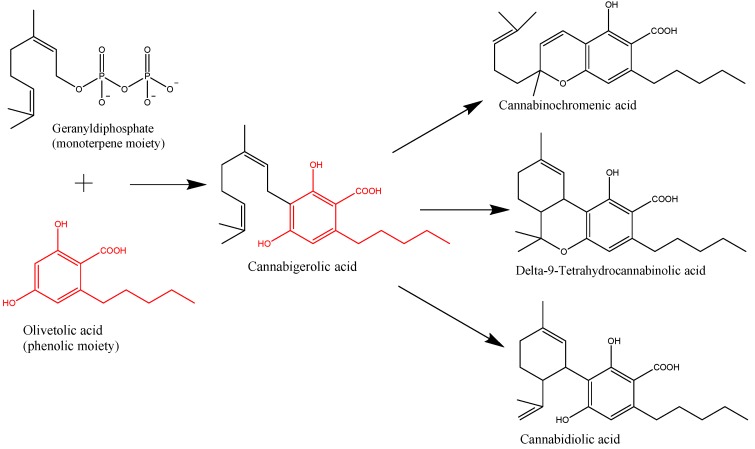
Key steps in the biosynthesis of the main phytocannabinoids from *Cannabis*.

Cannabinoids are terpenophenolics comprising a diphenol and a monoterpene moiety. The synthesis of the former part occurs via the polyketide pathway by the stepwise condensation of three malonyl-Coenzyme A molecules with hexanoyl-Coenzyme A, in order to yield olivetolic acid [18]. The monoterpene unit, geranyl-diphosphate, results from the head-to-tail condensation of geranyl-diphosphate and dimethylallyldiphosphate through the non-mevalonate pathway. Subsequently, olivetolic acid undergoes prenylation by geranyl-diphosphate (Figure 1). The product of this synthesis, cannabigerolic acid, is the key metabolic intermediary of cannabinoid biosynthesis [19]. It represents the substrate of three enzymes: tetrahydrocannabinolic acid synthase convertingcannabigerolic acid to Δ9-THCA [16], cannabidiolic acid synthase yielding cannabidiolicacid [20], and cannabinochromenic acid synthase producing cannabinochromenic acid [21]. Recent research could identify in planta both the acidic forms of the cannabinoids [22] as well as the decarboxylated forms (THC, CBD, cannabichromene, cannabigerol, cannabinol)—albeit in much lower amounts [23]. Non-enzymatic decarboxylation of these compounds is promoted by heating (during smoking or baking), sunlight, and storage. It is believed that the high number of diverse cannabinoids identified so far from *Cannabis* occur due to non-enzymatic modifications [24]. Studies performed on laser-microdissected capitate-stalked trichomes could suggest that cannabinoids are also present in the multicellular stipes of capitate-stalked hairs in addition to the secretory portion of the trichomes [23]. The exact contribution of the stalk cells to the biosynthesis of cannabinoids and the source of these compounds in the stems remains to be elucidated.

## 2. Pharmacology, Toxicology, and Route of Administration

Cannabidiol is the second most abundant type of cannabinoid, showing higher concentrations than THC in many cannabis strains [25]. Compared to THC, CBD has non-psychoactive effects, an advantage for clinical applications of the anti-tumor benefits [26].

### 2.1. Pharmacokinetics

The pharmacokinetics of CBD depends on its pharmaceutical formulation and on its routes of administration [27]. It is a highly lipophilic compound, having a poor oral bioavailability (around 6%) [28,29]. The absorption following inhalation is similar to the intravenous one, having a peak plasma concentration obtained within 3–10 min and being higher than the one obtained after oral administration (the bioavailability of inhaled CBD being around 31%) [30,31]. The explanation for this variable absorption lies in the fact that after oral administration CBD suffers an intense first pass metabolism; thus, the inhalation route can avoid or reduce the extent of this first pass metabolism [32]. Also the transdermal formulation can surpass the first pass metabolism [33]. Plasmatic levels can be increased if CBD is administered orally with food or when a meal is consumed after its administration, as lipids can increase its absorption [34].

CBD is rapidly distributed in the lungs, heart, liver, and brain, and to the less vascularised tissues. It has a high distribution volume and can accumulate in adipose tissue in patients under chronic treatment, having the risk of a prolonged activity (several weeks after administration) [28,35]. Thus, CBD distribution is influenced by age, body size, composition, and the permeability of the blood—tissues barriers [27]. Due to its lipophylic structure, CBD is able to cross placenta and also to arrive in breast milk. Peak plasma concentrations and area under the curve were reported to be dose-dependent in human studies with a T_max_ (time taken to reach the maximum concentration) of between 1 and 4h [34].

**Figure 2:**
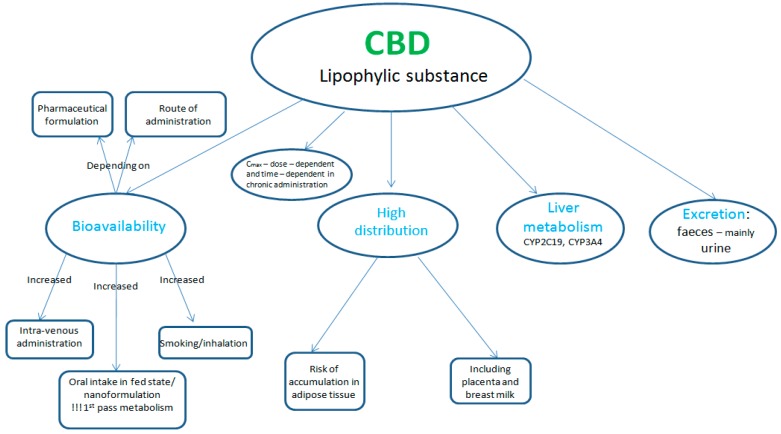
Characteristics of cannabidiol’s (CBD) pharmacokinetic profile.

Further, CBD is metabolised mainly in the liver, by the main isoenzymes CYP2C19 and CYP3A4, and to a lesser extent by CYP1A1, CYP1A2, CYP2C9, and CYP2D6 [36]. After its hydroxylation to 7-hydroxy-cannabidiol (7-OH-CBD) and further metabolism, its metabolites (mostly with unknown pharmacological activity) will be predominantly excreted in faeces and to a lesser extent in the urine [28]. Cannabidiol has a long terminal elimination half-life, the average half-life being around 24h ± 6h after intravenous administration and around 31 ± 4h after inhaled administration. After repeated intake of oral formulations, CBD elimination half-life was reported to vary between two and five days [37] (Figure 2). All these pharmacokinetic properties are important to be considered before CBD is administered as an anti-cancer agent.

### 2.2. Pharmacodynamics

Cannabidiol is a non-psychoactive cannabinoid, compared with THC, the psychoactive compound which produces the main side effects of cannabis [38,39]. Compared with THC, which is a partial agonist of cannabinoid receptor 1 (CB1, located mainly in the central nervous system, but also present in organs, tissues, and peripheral nervous system) and CB2 (expressed in immune tissues, gastrointestinal tract, and in low concentrations in the central nervous system) receptors of the endogenous cannabinoid system, CBD has a weak affinity for the sites of the receptors (the orthostatic ones). Moreover, it was reported to possibly inhibit THC binding to CB1 through other mechanisms [40,41,42].

The main physiological function of the endogenous cannabinoid system consists of inhibiting the release of other neurotransmitters (acetylcholine, dopamine, histamine, serotonin, glutamate, GABA, etc.) in the nervous system, usually by stimulating CB1receptors [30]. Cannabidiol presents a complex mechanism of action consisting of: weak blocking of CB1 receptors; an inverse agonist of CB2 receptors;stimulation of vanilloid receptors type 1 (TRPV1—transient receptor potential vanilloid 1) and type 2 (TRPV2—transient receptor potential vanilloid 2); increasing the concentration of anandamide (by blocking its hydrolysis), a fatty acid neurotransmitter (its effects being mediated through CB1 receptors in CNS and CB2 receptors in the periphery); stimulation of endogenous adenosine signalling (by binding to equilibrative nucleoside transporter-1); inhibition ofG-protein coupled receptor 55 (GPR55); and the stimulation of the 5-HT_1a_ (serotonin receptor 1A) receptor, PPARγ (nuclear peroxisome proliferation activated receptor γ) and glycine receptor subtypes [26,31] (Table 1).

### 2.3. Pharmacological Actions and Indications

In addition for its anti-tumor properties [51], CBD has been reported to induce the following effects: analgesic [52], neuroprotective [53], antiemetic [54], anticonvulsivant [55], anti-inflammatory [55], and antispasmodic [56].Thus, studies have shown its therapeutic potential not only in different types of malignant disorders [51], but also in the treatment of: epilepsy [57]; nausea and vomiting or other side effects caused by cytostatic therapy [54]; spasticity as well as other symptoms like tremor, bladder dysfunction, disease progression, inflammation, cognition in multiple sclerosis [58]; neuropatic and chronic pain [59]; spinal cord injury [60]; Parkinson’s and Alzheimer’s disease [61]; post-traumatic stress disorder and anxiety [62]; schizophrenia [63]; pulmonary disease [64]. Other therapeutic uses of CBD might be in the treatment of cannabis and tobacco addiction, although much more research is needed [65].

### 2.4. Toxicology

Cannabidiol has been reported to have low toxicity, is generally well tolerated, and has a good safety profile, although related studies are currently limited. It can interact with other co-administered drugs, causing different side effects depending on the interaction [65]. For the moment there are no reported risks of potential physical dependence (withdrawal and tolerance) or potential abuse [66,67,68].

Cannabidiol was reported to induce mainly fatigue and somnolence. On the other hand, THC was reported to induce dose-dependent performance (cognitive and psychomotor) impairment, as well as to increase anxiety, psychotic symptoms, heart rate, and blood pressure, and to alter perception. By mixing CBD with THC, the side effects of THC were reduced [67,68].

There are few data regarding its safety profile in children (limited pharmacokinetic information) [44]. Due to limited studies, and the ability of CBD to cross the placenta and to arrive in breast milk, it is not recommended for use in pregnancy and lactation [27,67,68].

Caution should be taken when CBD is administered in patients with hepatic impairment (risk of accumulation) or when taken with other drugs, because inducers/inhibitors of CYP3A4 or CYP2C19 can decrease/increase its plasmatic concentration [27,69]. Moreover, it acts as a CYP1A1 inducer and as an inhibitor of P-glycoprotein-mediated drug transport, which can affect the plasmatic concentration of co-administered drugs [27,69] (Table 2).

Both cannabis and tobacco smoking can induce CYP1A2 metabolism (with addictive effects when they are smoked together), causing significant interactions for substances that are substrates of this enzyme [27].

There were case reports of delirium and hypomania when CBD was associated with dysulfiram (unknown mechanism) and mania after association with fluoxetine (possibly CYP2D6 mediated) [67,68].

### 2.5. Route of Administration and Dosage

Cannabidiol is present for the moment in three pharmaceutical formulations, such as: Sativex^®^, containing nabiximols (almost equal amounts of THC and CBD), Epidiolex^®^ (pure CBD), currently in Phase III trials, and Arvisol^®^ (oral tablet containing pure CBD), currently in Phase I trials for the treatment of schizophrenia and epilepsy [66].

Sativex^®^ is an oromucosal spray, 100 µL = 2.7 mg THC + 2.5 mg CBD, obtained from *Cannabis sativa* L. It is suggestedto be used as a symptomatic treatment (in combination with other current anti-spasticity medicine) in adults with moderate-to-severe spasticity caused by multiple sclerosis, in case of failure of previous treatments. It requires up to two weeks of titration period to achieve an optimal dose and its administration should be associated with food intake. Nabiximols can be used once (afternoon or evening) or twice a day (morning and evening), the average dose being 8 sprays/day (maximum 12 sprays/day), with a minimum 15 min gap between administrations. After 4 weeks of treatment, the specialist physician should evaluate the patient’s response to the treatment. It is not recommended to be used in patients less than 18 years old and with caution in the elderly. It is contraindicated in case of hypersensitivity to cannabinoids, known/suspected history/familial history of schizophrenia or other psychotic illnesses, or severe cardiovascular diseases [67].

Epidiolex^®^ is an oral solution containing 100 mg/mL of CBD, which is suggested for the treatment of seizures associated with Lennox–Gastaut syndrome or Dravet syndrome in patients older than 2 years. The starting oral dose is 2.5 mg/kg, administered twice daily and which can be increased after one week at 10 mg/kg/day, the maximum dose being 20 mg/kg/day (divided into 2 administrations). Side effects of somnolence, sedation, hepatotoxicity, suicidal behavior, ideation (being recommended to monitor the patients), decreased appetite, diarrhea, fatigue, asthenia, sleep disorder, insomnia, and an increased risk of infections were reported [68].

### 2.6. Cannabidiol and Hepatotoxicity: A Debate

The literature presents controversial studies regarding the hepatotoxic potential of CBD. On the one hand, Mallat et al. have shown that activation of the body’s endocannabinoid system, specifically the CB2 receptor, is therapeutically beneficial in the treatment of many liver diseases. Moreover, studies have shown that CBD can help fight cirrhosis by kill hepatic stellate cells [70]. Other studies in the field have also underlined that CBD can reduce the inflammatory signalling pathways, thus limiting damage caused by cirrhosis [71]. In line with these findings, Yang et al. have shown that CBD prevents alcohol-induced oxidative stress and autophagy [72]. According to Ashino et al., cannabinoids inhibit the enzymatic activity of CYP1A, thus they have the ability to reduce the risk of liver toxicity [73]. In preclinical studies-animal models, CBD has been shown to be effective in restoring liver function in liver injury [74].

From a different viewpoint, Ewing et al. have demonstrated that despite the many beneficial effects of CBD, it may possess a risk for liver toxicity [75]. This research was tested in the acute and sub-acute phase. In the acute phase 0; 246; 738 or 2460 mg/kg of CBD was delivered to male B6C3F1 mice and observed for 24 h. The mice on the highest dose indicated a significant increase in liver-to-body weight ratios and also in total bilirubin values. In the sub-acute phase, the eight-week-old mice delivered orally daily doses of 0; 61.5; 184.5; or 615 mg/kg for 10 days. 75% of the mice instantly died at the 61.5 mg/kg dose and the rest of mice, at the same dose, present overt toxicity, which is manifested as profound lethargy, loss of appetite, and body weight loss [75].

## 3. Anticancer Effects of CBD in In Vitro and In Vivo Studies

**Figure 3:**
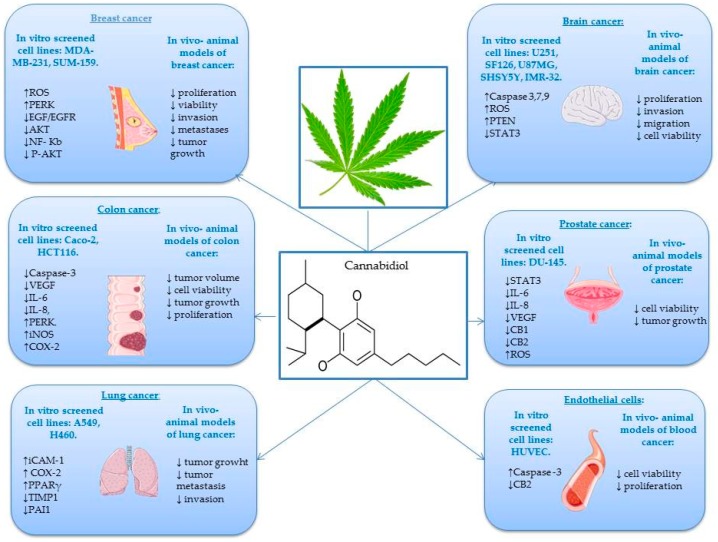
In vitro-in vivoanticancer mechanism of CBD in different types of cancerand the related effects on proliferation/apoptosis/gene expression/oxidative stress/tumor growth [76,77,78,79,80,81,82,83,84,85,86,87,88,89,90,91,92,93,94,95,96,97,98,99,100,101,102,103,104,105,106,107,108,109,110,111,112,113,114,115,116,117,118,119].

An increased number of studies have demonstrated that CBD and other structurally related cannabinoids present anticancer potential both in vitro on various cell lines (Table 3) as well as in vivo in a multitude of animal models [76]. As previously mentioned, CBD belongs to the cannabinoid family and is a non-psychoactive compound that binds to specific G-protein-coupled receptors [77]. Particularly, CBD is able to interfere with different stages of the tumor process, it can inhibit cancer cell migrations and adhesions, and exerts anti-proliferative, pro-apoptotic, and anti-invasive effects [78,79,80]. Since the first study of Munson et al. in 1975 on the in vitro and in vivo anti-proliferative potential of CBD, clinical use has gradually grown year after year. Cannabidiol presents chemo-preventive effects in some types of cancer, such as breast, lung, colon, prostate, skin, and brain [81,82,83]. After activating the expression of various genes, proteins, enzymes, and signaling pathways, CBD in different forms and concentrations plays a key role in different complex mechanisms that have as a final result the blocking of cancer initiation, progression, and metastation in different types of cancer [84] (Figure 3).

**Lung cancer** is one of the most common causes of cancer deaths. According to the World Cancer Research Fund International, in 2018 there were two million new cases of lung cancer detected worldwide [85]. In Europe, Hungary had the highest rate, followed by Serbia and France. The most frequent risk factor for lung cancer is tobacco smoking, which is the cause of 90% of lung cancers [86]. Ramer et al. demonstrated that CBD caused inhibition of A549 cell invasion.The mechanism was assigned to a decreased secretion of plasminogen activator inhibitor-1 (PAI-1), which is responsible for the anti-invasive action [87]. In another study, the same group have also reported that PAI-1 plays an important role in the anti-metastatic potential of CBD. The anti-invasive effect was determined by a modified Boyden chamber assay using 1 µM CBD and 72 h incubation period, whereas the PAI-1 expression (a key factor for tumor invasion and metastasis; a high concentration of PAI-1 is considered a poor prognostic factor in many types of cancer, such as lung, colorectal, gastric, and breast) was determined by RT-PCRusing 1 µM CBD over a 48 h incubation period [88,89]. Inter-intracellular adhesion molecule (ICAM-1) plays an important role in the interaction between lymphokine-activated killer cells and cancer cells. In lung cancer, the application of CBD resulted in an upregulation in the expression of ICAM-1 event directly correlated with the prevention of metastasis of cancerous cells beyond the tumor site [64]. Ramer et al. demonstrated that 10 mg/kg/day of CBD reduces A549 and H460 lung cancer cell line viability in vivo in an animal model using athymic nude mice. The cellular mechanisms induced by CBD presume an up-regulation of cyclooxygenase (COX-2) and PPAR-γ, in vitro as well as in vivo [90].

The number of **melanoma** cases has permanently increased over the past few years compared to other types of cancer [91]. A recent study conducted by Simmerman et al. reported that CBD represents a potentially new therapeutic agent for malignant melanoma. In this study, murine B16F10 tumors were implanted in 8–12-week-old male mice and mice were treated with cisplatin (5 mg/kg/week) and CBD (5 mg/kg twice per week). Results have shown that the group treated with CBD exhibited similar behavior to the group treated with the consecrated anticancer drug cisplatin, namely, significantly reduced melanoma tumor growth and increased survival time and quality of life [92,93,94].

**Breast cancer** is the primary cause of death among women and is the second most common cancer overall [95]. There are many risk factors, such as age, history of breast cancer in the family, genetics (women who carry the breast cancer gene 1 (BRCA1), breast cancer gene 2 (BRCA2), and tumor protein p53 (TP53) genes), contraceptives, and lifetime duration of breastfeeding [96]. Using MDA-MB-231 and MCF-7 cells, Shrivastava et al. have explained that CBD induces cell death in selected cancer cells. Among the mechanisms, they have suggested that CBD induces endoplasmic reticulum stress and apoptosis by inhibiting the AKT/mammalian target of rapamycin (mTOR) signaling (5 µmol/L CBD for 24 h) and enhances reactive oxigen species (ROS) generation for selected breast cancer cells (5 µmol/L CBD for 12 h) [97]. Other studies have demonstrated that CBD inhibits the growth of different breast tumor cell lines (MCF-7, MDA-MB-231) with an IC_50_ value of about 6 µM and exhibits significantly lower potency in non-cancer cells [57]. Recent research showed that CBD induces apoptosis in two different human breast cancer cell lines: T-47D and MDA-MB-231. These effects were observed by MTT assay, DNA fragmentation, and ELISA apoptosis assays. The MTT screening showed that the IC_50_ values were 2 µM (MDA-MB-231 cells) and 5 µM (T-47D cells). At the same time, both cell lines showed improved nuclear localization of PPARγ following treatment with 1–7 µM CBD for 24 h. Moreover, treatment with CBD led to an interaction between PPARγ, mTOR, and cyclin D1 to the advantage of apoptosis induction [98]. In 2014, Elbaz et al. studied the anti-tumor mechanism of CBD: they showed that it inhibits epidermal growth factor-induced proliferation. The study concluded that CBD (3, 6, and 9 µM) can be used as a novel option to inhibit growth and metastasis of aggressive breast cancer cells [99]. McAllister et al. reported the efficacy of 1.5 µM CBD in the development of breast cancer metastasis in vivo as well as in vitro, using cell proliferation and invasion assays, flow cytometry and Western blotanalysis [100].

**Colon cancer** is the second most common cause of cancer patient mortality all over the world. Moreover, colorectal cancer is the third deadliest cancer in the United States [101,102]. Aviello et al. showed that CBD exerts a significant antiproliferative effect in two colorectal carcinoma cell lines (Caco-2 and HCT116). They used in vivo experiments, and the cells were treated with 0.01–10 µM CBD for 24 h in a male Institute of Cancer Research (ICR) mouse model of colon cancer. Treatment with just 1 mg/kg CBD significantly reduced aberrant crypt foci (ACF) polyps and tumors in male mice. Moreover, results have shown that by starting from this dosage, CBD presented an optimal chemo-preventive effect. The protective effect on colon cancer was associated with up-regulation of Caspase-3 [103]. Kargl et al. pointed out that GPR55 is implicated in the migratory behavior of HCT116 colon cancer cells and plays an important role in the prevention of metastasis. For this assertion, they used adhesion and migration assays. The GPR55 antagonist CID 16020046 (1, 2.5, 5 µM), CBD (1, 2.5 µM), a putative GPR55 antagonist and GPR55 small interfering RNA (siRNA) were used to block GPR55 activity of HCT116 colon cancer cells [104]. Another study has demonstrated that HCT116 and DLD-1 colorectal cancer cell cultures treated with different concentrations of CBD (0–8 µM) presents phenomena of apoptosis. The mechanism refers to the regulation of many proteins, of which Noxa showed significantly higher expression [105]. A recent study investigated the effect of CBD on the CT26 colon cancer line, in vivo, in an animal model using male BALB/c mice. Results have shown that 1–5 mg/kg CBD has an encouraging effect on reducing colon cancer growth and decreasing tumor size. These favorable effects may be due to growing activity of antioxidant enzymessuperoxide dismutase (SOD) and glutathione peroxidase (GPX) [106].

**Prostate cancer** is one of the most common types of cancer in men [107]. A recent study has demonstrated that CBD is a novel modulator of exosome and micro vesicle (EMV) release in several cancer cell lines (EMV plays an important role in limiting tumor growth). This study showed that 1–5 µM of CBD significantly reduced growth of PC3 prostate cancer cells [108]. Petrocellis et al. tested both in vitro and in vivo the effect of CBD against LNCaP prostate cancer cells. Results suggested that the anticancer mechanism involves the stimulation of intrinsic pathways of apoptosis. In vivo, CBD (1–100 mg·kg^−1^) significantly inhibited prostate cancer cell viability in an animal model using male MF-1 nude mice [109]. Sharma et al. worked on the evaluation of the antitumor activity of CBD in LNCaP and PC3 prostate cancer cell lines. The result indicated that 20–60 µg/mL CBD is a potent inhibitor of cancer cell growth. The effect was evaluated by ELISA assay and flowcytometry [110]. Another investigation into the effect of CBD on LNCaP prostate carcinoma cells demonstrated that 5–15 µM CBD inhibits cancer cell growth. The study concluded that, regarding this cell line, the pro-apoptotic activity of CBD was phosphatase-dependent. It is worth noting that the anti-tumoral effects of many cannabinoids include modulation of intracellular kinase [111].

**Brain cancer** is identified as one of the most terrible forms of cancer due to several impediments [112]. Massi et al. demonstrated that CBD led to a concentration-related inhibition of the U87 human glioma cell viability after 24 h of incubation with CBD 5–10 µM. Moreover, the phytocompounds inhibited the growth of U373 and U87 human glioma cell lines implanted in athymic female CD-1 nude mice [113]. Recently, Singer et al. have demonstrated that CBD inhibits the viability of 3832 and 387 glioma stem cell (GSC) lines and induces apoptosis by the production of ROS. Moreover, the in vivo treatment of intracranial GSC-derived tumors with CBD (15 mg/kg) inhibited tumor cell proliferation, activated pro-apoptotic caspase-3, and significantly prolonged the survival of mice. Even though GSCs adapted to CBD treatment, this fact has been suppressed by combining therapy of CBD with small molecule modulators of ROS (vitamin E). Taken together, these data suggest that a combination of CBD with vitamin E regulates ROS levels. This can represent a promising therapeutic model for glioblastoma management [114]. Furthermore Marcu et al. demonstrated that 0.4µM CBD inhibits the growth of different glioblastoma cell lines (U87-MG, U251, and SF126). They concluded that CBD is a more potent inhibitor of glioblastoma cell growth than THC [115]. The role of the transient receptor potential (TRP) channel is the regulation of cellular proliferation and differentiation. Nabissi et al. have shown that CBD represents a specific ligand for TRPV2; thus, CBD works as a TRPV2-selective activator by intensifying Ca^2+^ influx in U87MG cells with an IC_50_ of 22.2 μM. Thereby, CBD could be used as a promising therapeutic agent against GBM cancer cell lines [116]. Solinas et al. proved that 1–10 μM of CBD induces apoptosis and inhibits human umblilical vein endothelial cell (HUVEC) migration using ELISA assay and angiogenesis array kit [117]. Another research group demonstrated that the cannabinoid loaded microparticules was shown to enhance apoptosis and decrease cell proliferation in glioma U87MG cells. The microparticules which contain only CBD (6.7 mg) was administeredin vivo on experimental athymic nude mice [118]. Alharris et al. demonstrated that 10 µM CBD induces apoptosis in neuroblastoma SH SY5Y and IMR-32 cell lines through activation of serotonin and vanilloid receptors, also significantly reducing cancer cell migration and invasion in vitro [119].

## 4. Immunomodulatory Effects of CBD

One of the struggles of cancer treatment consists in the possibility of activation of the immune system against the tumor. In recent years scientists’ efforts were focused on developing therapies that target tumor immunity [120,121]. Immunotherapy is considered a distinct category from classic cytotoxic therapies used for cancer treatment [121]. Based on the idea that it would be great to find a natural candidate for an anticancer agent that can on the one hand “kill” the cancerous cells via different mechanisms and pathways and on the other hand stimulate the immune system, this review also examines this aspect. Among various therapeutic effects, CBD also possesses immunomodulatory potential [122].

The stimulation of cannabinoid receptors (CBR) can lead immune cells to regulate the DNA binding of various nuclear factors, an effect mainly triggered by down-regulation of cyclic adenosine monophosphate (cAMP) formation [123]. Cyclic adenosine monophosphateanalogues can produce inhibition or stimulation in a dose-dependent manner of the immune responses and can affect cannabinoids’ effects on T-cell-dependent production of antibodies [123]. In contrast to THC, the non-psychoactive CBD was reported to have a low affinity for CBR [123].

Both in vitro and in vivo models have been used in order to evaluate the CBD effects on T-cells and macrophages. Results indicated that CBD has the ability to alter the reactivity of the immune system’s cells [124].

Cannabidiol has been reported to decrease the production of T-helper 2 cytokines such as IL-10, which is well known to play an important role in humoral immunity [125]. Furthermore, Malfait et al. showed that i.p. or s.c. administration of CBD to mice decreased tumor necrosis factor α (TNFα) and also reduced interferon gamma (IFN-γ) production [126]. Moreover, CBD was shown to reduce IL-1 and TNF in human peripheral blood mononuclear cells [127]. The TNFα and IL-1β expression in macrophages is regulated by the nuclear factor κB (NFκB), which augments the expression of anti-apoptotic molecules in cancer cells, leading to resistance of tumor cells to existing chemotherapies [128]. Reduction of TNFα and IL-1β expression in macrophages by CBD suggests its therapeutic anticancer potential.

Macrophages have an important role in innate and adaptive immunity and are one of the main producers of IL-12 [129]. Sacerdote et al. showed that both in vitro and in vivo administration of CBD elicited an increase in IL-12 production and a decrease in IL-10, respectively [130]. A potent antitumor cytokine, IL-12 is able to provoke tumor regression and reduce the formation of distant metastasis following systemic or peritumoral administration [130].

Another study performed on splenocytes derived from CB1^−/−^/CB2^−/−^ mice showed that CBD administration caused suppression of IL-2 and IFN-γ expression and proliferation, suggesting an inhibition of T-cell function [131].

According to Carrier et al., CBD possesses immuno-suppressive effects through the enhancement of endogenous adenosine signaling [132]. The authors showed that CBD acts as a inhibitor of adenosine and thiamine uptake by inhibiting the equilibrative nucleoside transporter-1 (ENT-1) [132]. Due to these effects, CBD arises as an interesting compound in cancer patients’ therapy [131]. During tumor pathogenesis, the purine nucleoside, adenosine, is secreted by cancer and immune cells under metabolic stress and hypoxia [130]. Adenosine binding to A2A receptor stimulates IL-4 and IL-10 release that enhanced tumor cell growth by triggering the suppression of the antitumor immune response [130].

In a study performed on splenocytes, CBD treatment induced a reduction of IL-2, IL-4, and IFN-γ production. Moreover, when tested on mice prior to ovalbumin sensitization, CBD induced a significant inhibition of antigen-specific antibody production, indicating an effect on the suppression of humoral immunity [133].

In a recently published paper, Jensen et al. evaluated the immune gene expression following treatment with CBD using a zebrafish model that resemblances the human genome by around 70%. The authors concluded that CBD modulated the immune genes differently, up-regulating IL1B and IL17A/F2 and down-regulating transforming growth factor beta, alpha (TGFBA), S100A10B (S100 calcium-binding protein A10), immunoglobulin heavy constant Mu (IGHM) and CD4-1 [126]. The non-psychoactive component of *Cannabis sativa* L., CBD, proved to have a modulatory effect on tumor immunity, suggesting its therapeutic potential in cancer treatment. The compound is considered to be a component of the formulation known as “medical cannabis,” which is currently used in some countries [129]. Further studies are required in order to fully understand all the mechanisms involved in CBD antitumor activity.

## 5. CBD in Inflammation-associated Carcinogenesis

Although for all higher organisms inflammation is the most competent defensive response of the innate and adaptive immune system, when it becomes chronic it can eventually cause organ dysfunction and structural impairment. Various studies have shown that sphingolipids participate in the structural preservation of cell membranes and mediate cellular functions specifically: migration, proliferation, and apoptosis. Therefore, they are prone to regulate the fate of the cell and consequent onset of inflammation and cancer [134]. Sphingosine-1-phosphate (S1P) is an extracellular ligand for G protein-coupled receptor sphingosine-1-phosphate receptor 1 (S1PR1), and it can activate signal transducers and activators of transcription 3 (STAT-3), a pro-survival pathway implicated in the conversion of inflammation to oncogenesis. The cellular, extracellular, and tissue concentrations of S1P are regulated by its irreversible degradation by sphingosine-1-phosphate lyase (SGPL1). This key enzyme appears to be an auspicious drug target for the design of immunosuppressants [134].

Schwiebs et al. have shown that, based on the initiating cellular S1P source, the pathophysiology of inflammation-induced cancer and cancer-induced inflammation evolve through separate, observable molecular stages. The presence of two different mechanisms of carcinogenesis production was observed in a model of compartment-specific SGPL1 depletion in immune cell compartment and tissue cell compartment. In the tissue cell section, they noted fast tumor growth with particular modulation of the tumor microenvironment, and chronic, complex inflammation with succeeding, but relatively delayed carcinogenesis, in the immune cell section [134]. The theory that inflammation may lead to the commencement of cancer is rational considering the following common events: increased genomic injury and DNA synthesis, cellular multiplication, disruption of DNA restoration pathways, the promotion of angiogenesis, and inhibition of apoptosis [135]. Therefore, a potentially anti-inflammatory compound may have a chemopreventive effect.

Summarizing these aspects, chronic inflammation increases the probability of different types of cancer, suggesting that abolishing inflammation may represent a well-founded strategy for cancer prevention and therapy [135]. These findings suggest that CBD’s anti-inflammatory action highlights it as a potential anticancer agent worthy of clinical consideration for cancer therapy. The dual therapy consisting of an anti-inflammatory agent and conventional anticancer drugs may improve patient prognosis and metastasis [135].

## 6. Anti-angiogenic Effects of CBD

As already described above, the multi-target effect of CBD includes anti-proliferative and pro-apoptotic activities, and as more recently described, anti-angiogenic properties [117]. Angiogenesis, the formation of new blood vessels from preexisting ones, is an essential multistep generating growth, invasion of cancer cells, and metastasis. This process can be modulated by targeting several key factors, by inhibiting growth factors, such as vascular endothelial growth factor (VEGF), integrins, angiopoietins, or by activating inhibitory effectors as thrombospondin andinterferons [117].

Cannabinoids were found to be responsible for down-regulating VEGF receptors in different cancer types. Apoptosis of endothelial cells was observed by activating CB receptors [136], and a reduction of pro-angiogenic factors was also observed [137].

So far, few studies have investigated the effects of CBD as an angiogenesis modulator. Solinas et al. discovered strong anti-angiogenic effects of CBD, both in vitro, reducing growth, migration, and invasion of HUVEC cells, and in vivo, using the Matrigel sponge assay in mice. Multiple mechanisms in modulating angiogenic factors relatingto impaired angiogenesis were observed [138].

An interesting difference compared to the results obtained for cannabinoids in general was that CBD did not induce either apoptosis or necrosis on HUVEC cells at high concentrations (12 µM), but it did induce endothelial cell migration. The anti-migration activity was supported by the molecular modulation of several factors. Cannabidiol inhibits matrix metalloproteinase-2, 9 (MMP2, MMP9) and tissue inhibitor of metalloproteinases 1 (TIMP1), thus impairing cell motility and invasion, suppresses the activity of urokinase-type plasminogen activator (uPA), and serpinE1/plasminogen activator inhibitor-1 (PAI1), which are involved in extracellular matrix degradation. By inhibiting chemokineligand 16 (CXCL16) and IL-8, cell motility and network formation are impaired, whereas down-regulating endothelin-1 (ET-1), platelet-derived growth factor subunit A (PDGF-AA), and VEGF expression, micro vessel density is reduced [117]. Vascular endothelial growth factor was found to be down-regulated by CBD in glioma and prostate cancer. Another study indicated that CBD also inhibits HIF-1a (the regulatory subunit of the hypoxia-inducible transcription factor) in U87-MG glioma cells, therefore suggesting its involvement in cell survival, motility, and angiogenesis in a hypoxic environment [138].

A more recent publication explored how CBD could inhibit angiogenesis in colon cancer, and it was shown that it significantly decreased the level of proinflammatory cytokines IL-6 and IL-8 and increased the activity of malonaldehyde (MDA), an antioxidant enzyme. Others found that CBD reduces the levels of an mTOR substrate and STAT5, inducing vasorelaxation, thus contributing to the underlying mechanism of the anti-angiogenic effect in human endothelial cells [138,139].

Another recent study evaluated the effect of CBD in breast cancer and found that it can modulate the tumor microenvironment by reducing the recruitment of macrophages, which leads to angiogenic inhibition [140].

Next to its low toxicity and the non-psychoactive activity, the anti-angiogenic properties and the multi-target anti-tumor effects indicate that CBD is an interesting candidate for anticancer clinical applications [138].

## 7. Clinical Evidence of the Anticancer Effects of CBD

Today, CBD has become extraordinarily popular around the world. It is now accessible in a growing number of products with different administration modes [141]. There are many types of dietary supplements like capsules, gummies, tinctures, and oils. For topical administration, creams, lotions, and ointments are frequently employed, but the most common is CBD oil. Oil has become a preferred mode of administration for many CBD users for multiple reasons. The foremost motive is that the oil is very easy to administer; also, it allows consumption of a high dose of CBD in a lightly ingestible form [142].

Currently, there are hundreds of active manufacturers and sellers of CBD, and their number is rapidly growing, because CBD is capable, as shown by important studies in the field, of inhibiting the development of an increased number of cancer cells both in vitro and in vivo [143].

Information collected to date in relation to the anticancer effects of CBD are nearly completely limited to preclinical studies conducted on cell lines and also on animal models [84]. Although there is a lot of literature on preclinical in vitro and in vivo studies that describe the anticancer mechanism of CBD on various types of cancer, the number of clinical trials which have as a research theme the study of the effect of CBD on different types of cancer is limited [144]. To discover the full scope of its positive effects on cancer, more human studies are needed to investigate the toxicological parameters. The risks about the long administration’s effects are unknown, especially for children. Drug interaction studies are necessary from both a therapeutic and a safety viewpoint. To determine the risks and also the benefits by the help of clinical trials remains desirable, but this will take a longtime and require a big budget [145].

In terms of clinical trials, CBD was most studied for glioblastoma. A clinical trial that analyzed the effect of CBD as a single agent for solid tumor was conducted in 2014 (clinical trial: NCT02255292). Another placebo-controlled phase II clinical trial analyzed the effect of the combination of THC and CBD with adjuvant chemotherapy with temozolomide for patients with glioblastoma, and reported positive results using this approach (clinical trial:NCT01812603) [146].

An outstanding example is the use of CBD products for the self-medication of cancer with the aimof thoroughly curing it. In 2018, Sulé-Suso et al. reported an important case: an 81-year-old man, who was diagnosed with lung adenocarcinoma in 2016, refused chemotherapy and radiotherapy and began self-administration of CBD oil. He started first with two drops twice daily for a week and then nine drops twice daily for a month. One month later, a significant reduction on the number and size of mediastinal lymph nodes was observed on CT scan [147].

In 2018, a clinical trial with 119 cancer patients was conducted over a four-year framed period. Patients were given CBD oil three days on and three days off, with an average dose of 10 mg twice a day (max 30 mg for increased tumor mass). In this study, patients with different types of cancer (e.g., breast, prostate, and esophageal) were included. From 119 cancer patients, the most stunning case was a 5-year-old male patient with anaplastic ependymoma, a very rare brain tumor. The patient had all the standard treatments (surgery, chemotherapy, radiotherapy). He started the treatment with CBD oil in February 2016, and in December 2016 the relevant scans showed that tumor volume had decreased by approximately 60%. The other patients from this study have reported that the side effects completely disappeared, and although the duration of treatment was six months, many continued the treatment [148]. Stella et al. reported a case in which two patients were diagnosed with high-grade gliomas. In addition to chemotherapy and a drug regimen, CBD oil (ranging from 300 to 450 mg/day) was also involved. Both patients had admissible clinical and imaging responses as well as positive responses to treatment at periodic assessments [149]. Another case study presents an 81-year-old woman who was diagnosed with ovarian cancer in March 2017. The patient declined all interventions due to the treatment toxicity. She started with alternative therapy CBD oil (1 drop sublingually/day) and Laetrile tablets, which contain purified amygdalin (500 mg 4 times/day). After two months, CT imaging showed a dramatic decrease in the size of the tumor [150]. In a recent comprehensive review, Dumitru et al. also observed that the phase II clinical trials results indicate an increased life span for patients treated with CBD. Currently CBD is in a 180-day clinical trial-randomized double-blind, placebo-controlled parallel multi-center study including 160 patients for the evaluation of the effect of the cannabinoid combined with standard treatment for patients that suffer from multiple myeloma, glioblastoma multiform, and GI malignancies (clinical trial: NCT03607643) [151].

All these clinical cases prove that CBD demonstrates promising results, as it has undeniably helped different types of cancer patients. Although the literature describes the results of some clinical trials that include the pharmacokinetics and pharmacodynamics of CBD, there is an urgent need for further clinical trials to gain a complete picture of the effect of this molecule within the human body.

## 8. Conclusions

This review provides an update on the phytochemistry of CBD. Moreover, it highlights important aspects of the pharmacokinetics, pharmacodynamics, toxicology, route of administration, and dosage of this bioactive molecule. An in-depth screening of the literature led to the conclusion that preclinical studies (in vitro and in vivo) have shown that CBD possesses a very complex mechanism of action through which it can inhibit tumor formation and propagation in different types of cancer. However, despite the large number of preclinical studies, further research is needed because at this point there is not yet enough clinical evidence to prove that CBD can safely and effectively treat any or one particular type of cancer in humans. To date, most clinical positive outcomes following treatment with CBD have been reported for glioblastoma. However, all the data described above suggest that CBD is a promising candidate for an anticancer agent.

## Figures and Tables

**Table 1:** Receptor affinity.

Receptor	Effect	K_i_; EC_50_; IC_50_	References
CB1	Antagonist	K_i_ = 4350–4900 nM	[43,44]
CB2	Inverse agonist	K_i_ = 2860–4200 nM	[43,45]
GPR55	Antagonist	IC_50_ = 445 nM	[45]
TRPM8	Antagonist	IC_50_ = 80–140 nM	[46,47]
TRPV1	Agonist	EC_50_ = 1000 nM	[46,48]
TRPV2	Agonist	EC_50_ = 1250 nM	[46,48]
TRPV3	Agonist	EC_50_ = 3700 nM	[46,49]
TRPA1	Agonist	EC_50_ = 110 nM	[46,48]
PPARϒ	Agonist	EC_50_ = 20,100 nM	[50]

Legend: CB1—cannabinoid receptor 1; CB2—cannabinoid receptor 2; EC_50_—Half maximal effective concentration; GPR55—G-protein coupled receptor 55; IC_50_—half maximal inhibitory concentration; K_i_—Inhibitory constant binding affinity; TRPA1—transient receptor potential ankyrin 1; TRPM8—transientreceptor potential melastanin 8; TRPV—transient receptor potential vanilloid.

**Table 2:** Inducers, and inhibitors for the main isoforms of cytochrome P450 that are implicated in the metabolism of CBD [27,69].

CYP 450 – Isoenzymes	Substrates	Inducers	Inhibitors
CYP3A4	**CBD** Alprazolam Diazepam Amlodipine Simvastatine Atorvastatine Apixaban Rivaroxaban	Carbamazepine Fenitoine Phenobarbital	Eritromicine Claritromicine Verapamil Diltiazem Fluconazol Itraconazol
CYP2C19	**CBD** Clopidogrel Fenitoine Diazepam	Carbamazepine Rifampicine	Fluoxetine Fluvoxamine Ketoconazole Omeprazole
CYP1A2	Theophylline Clozapine Naproxen	**CBD** Tobacco smoke	Ciprofloxacine Ofloxacine Levofloxacine Amiodarone
P-Glycoproteine (intestinal absorption)	Loperamide Morphine Dabigatran Atorvastatine Simvastatine Paclitaxel Antraciclines	Carbamazepine Rifampicine Phenobarbital	**CBD** Ketoconazol Itraconazol Eritromicine Clarytromicine Propafenone Amiodarone

**Table 3:** Role of CBD among various cancer cell lines.

Type/Cancer Cell Line	Cell Line	In vitro	In vivo	Conc.	Conclusion	Ref.
Colorectal cancer	HCT116		√	0–8 µM 100 mg·kg^−^^1^	CBD induces apoptosis by regulating many pro- and anti-apoptotic proteins, and decreases tumor volume	[105]
Colorectal cancer	DLD-1		√	0–8 µM 100 mg·kg^−^^1^		
Colon cancer	CaCo-2 HCT116	√ IC_50_ = 0.67 µM	√	5 mg/kg	Reduced aberrant crypt foci (ACF) number of polyps and tumors	[103]
Colon cancer	CT26		√	5 mg/kg	CBD induces apoptosis, showed anti-angiogenesis and anti-metastatic effect	[106]
Colon cancer	HCT116		√	5 mg·kg^−1^	CBD reduces colon cancer cells	[104]
Prostate cancer	PC3		√	1–5 µM	CBD reduces exosome release.	[108]
Prostate cancer	LNCaP		√	1; 10; 100 mg/kg	CBD decreased cell viability and tumor growth	[109]
Prostate cancer	DU-145		√	20–80 µg/mL	CBD is a potent inhibitor of cancer cell growth and has lowest potency in non-cancer cells	[110]
Prostate cancer	LNCaP		√	20–80 µg/mL		
Prostate cancer	PC3 LNCaP		√	5–15 µM	CBD induces apoptosis	[111]
Lung cancer	A549		√	5 mg/kg	CBD decreased tumor growth	[87]
Lung cancer	H460		√	3µM	CBD decreased tumor metastasis	[88]
Lung cancer	A549		√	3 µM	ICAM-1 present an essential objective for CBD in executing its antitumorigenic function.	[64]
Lung cancer	A549 H460	√	√	3 µmol/L 5–10 mg/kg	CBD induces cancer cell apoptosis	[90]
Brain tumor	U87 U373		√	5–10 µM	CBD induces apoptosis through activation of serotonin and vanilloid receptors	[113]
Brain tumor	GSC	√ IC_50_ = 3.5 µM	√	15 mg/kg	CBD induces apoptosis through the production of ROS	[114]
Brain tumor	U251 SF126	√ IC_50_ = 1.1–1.3 µM		0.4 µM	CBD induces apoptosis and reduces cell viability and invasion	[115]
Brain tumor	U87MG	√		10 µM	CBD activates TRPV2 receptors to promote cancer cell death.	[116]
Brain tumor	U87MG		√	6.7 mg	CBD enhances apoptosis and decreases cell proliferation.	[118]
Brain tumor	SH SY5Y IMR-32	√		10 µM	CBD induces apoptosis and reduces cancer cell migration and invasion	[119]
Skin cancer	Murine B16F10 melanoma tumors		√	5 mg/kg	CBD reduces tumor size	[92]
Breast cancer	MDA-MB-231	√ IC_50_ = 6–10.6 µM	√	10 mg/kg	Decreased tumor growth	[51]
Breast cancer	T47D MDA-MB-231		√	10 mg/kg	Decreased tumor metastasis	[98]
Breast cancer	MDA-MB-231		√	5 mg/kg	CBD induces cancer cells apoptosis	[97]
Breast cancer	SUM-159	√	√	3–18 µM	CBD induces both apoptosis and autophagy-induced death in cancer cells	[99]
Endothelial cells	HUVEC	√	√	1–19 µM	CBD inhibited cell proliferations and exhibited potent antiangiogenic properties inhibiting cell invasion and migration	[117]

## Abbreviations

5-HT1A	Serotonin receptor type 1A
ACF	Aberrant crypt foci
AD	Alzheimer’s disease
Aᵦ42	Amiloid beta 42
BCE	Before Common Era
BRCA-1	Breast cancer gene 1
BRCA-2	Breast cancer gene 2
cAMP	Cyclic adenosine monophosphate
CB1, CB2	Cannabinoid receptor 1,2
CBD	Cannabidiol
CBR	Cannabinoid receptor
CD-1; 4	Cluster of differentiation 1; 4
CGS	Glioma stem cells
CID 16020046	Inverse agonist at the former orphan receptor GPR55
COX	Cyclooxygenase
CT	Computed tomography scan
CXCL16	Chemokine (C-X-C motif) ligand 16
DNA	Deoxyribonucleic acid
EAE	Experimental autoimmune encephalomyelitis
EC_50_	Half maximal effective concentration
EGF/EGFR	Epidermal growth factor/epidermal growth factor receptor
ELISA	Enzyme-linked immunosorbent assay
EMA	European Medicine Agency
EMV	Exosome and microvesicle
ENT-1	Equilibrative nucleoside transporter-1
ET-1	Endothelin-1
FDA	Food and Drug administration
GABA	Gamma-aminobutyric acid
GI	Gastro-intestinal
GPR55	G-coupled protein receptor-55
GSC	Glioma stem cell
GW9662	Potent antagonist of Peroxisome proliferator-activated receptor gamma
GPX	Glutathione peroxidase
HUVEC	Human umbilical vein endothelial cells
HIF-1a	Regulatory subunit of the hypoxia- inducible transcription factor
i.p.	Intraperitoneal
IC_50_	Half maximal inhibitory concentration
ICAM	Inter-intracellular adhesion molecule
ICR	Institute of Cancer Research
IFN-γ	Interferon Gamma
IGHM	Immunoglobulin heavy constant Mu
IL-2,6,8,9,12,IL-1A; 17A/F2	Interleukin 2, 6,8,9,12, 1A, 17A/F2,
iNOS	Inducible nitric oxide synthase
Ki	Inhibitory constant binding affinity
KPC	KRAS, p53, Cre mice
MDA	Malonaldehyde
MIP-1α,1ᵦ,2	Macrophage inflammatory protein 1α,1ᵦ, 2
MMP2,9	Matrix metalloproteinase-2, 9
mRNA	Messenger RNA
Mtor	Mammalian target of rapamycin
NFκB	Nuclear factor κB
n.d.	Not determined
PAI-1	Plasminogen activator inhibitor-1
PDGF-AA	Platelet-derived growth factor subunit A
PERK	Extracellular signal-regulated kinase phosphorylation
Phospho-Akt	Phosphorylated AKT
PPARγ	Nuclear peroxisome proliferation activated receptor
PSA	Prostate-specific antigen
PTEN	Phosphatase and tensin homolog
ROS	Reactive oxygen species
RT-PRC	Real-time polymerase chain reaction
s.c.	Sub cutaneous
S100A10B	S100 calcium-binding protein A10
S1P	Sphingosine-1-phosphate
S1PR1	Sphingosine-1-phosphate receptor 1
Serpin E1	Serpin Family E Member 1
SGPL1	Sphingosine-1 phosphate lyase
SiRNA	Small interfering RNA
SOD	Superoxide dismutase
STAT-3,5	Signal transducers and activators of transcription 3,5
TGFBA	Transforming growth factor beta, alpha
THC	Tetrahydrocannabinol
THCA	Tetrahydrocannabinolic acid
TIMP1	Tissue inhibitor of matrix metalloproteinases1
TNF-α	Tumor necrosis factor α
Tp53	Tumor protein p53
TRP	Transient receptor potential
TRPA	Transient receptor potentialankyrin
TRPV1	Transient receptor potential vanilloid type1
TRPV2	Transient receptor potential vanilloid type 2
TRPM8	Transient receptor potentialmelastatin 8
uPA	Urokinase-type plasminogen activator
VEGF	Vascular endothelial growth factor
VR1, VR2	Vanilloid receptor 1, 2
